# The GERtality Score: The Development of a Simple Tool to Help Predict in-Hospital Mortality in Geriatric Trauma Patients

**DOI:** 10.3390/jcm10071362

**Published:** 2021-03-25

**Authors:** Julian Scherer, Yannik Kalbas, Franziska Ziegenhain, Valentin Neuhaus, Rolf Lefering, Michel Teuben, Kai Sprengel, Hans-Christoph Pape, Kai Oliver Jensen

**Affiliations:** 1 Department of Traumatology, University Hospital of Zürich, 8091 Zürich, Switzerland; julian.scherer@usz.ch (J.S.); yannik.kalbas@usz.ch (Y.K.); franziska.ziegenhain@usz.ch (F.Z.); valentin.neuhaus@usz.ch (V.N.); michel.teuben@usz.ch (M.T.); kai.sprengel@usz.ch (K.S.); hans-christoph.pape@usz.ch (H.-C.P.); 2 Institute for Research in Operative Medicine (IFOM), University of Witten/Herdecke, 58453 Cologne, Germany; Rolf.Lefering@uni-wh.de

**Keywords:** geriatric trauma, scoring, polytrauma, ISS, AIS, geriatric patients, orthogeriatric

## Abstract

Feasible and predictive scoring systems for severely injured geriatric patients are lacking. Therefore, the aim of this study was to develop a scoring system for the prediction of in-hospital mortality in severely injured geriatric trauma patients. The TraumaRegister DGU^®^ (TR-DGU) was utilized. European geriatric patients (≥65 years) admitted between 2008 and 2017 were included. Relevant patient variables were implemented in the GERtality score. By conducting a receiver operating characteristic (ROC) analysis, a comparison with the Geriatric Trauma Outcome Score (GTOS) and the Revised Injury Severity Classification II (RISC-II) Score was performed. A total of 58,055 geriatric trauma patients (mean age: 77 years) were included. Univariable analysis led to the following variables: age ≥ 80 years, need for packed red blood cells (PRBC) transfusion prior to intensive care unit (ICU), American Society of Anesthesiologists (ASA) score ≥ 3, Glasgow Coma Scale (GCS) ≤ 13, Abbreviated Injury Scale (AIS) in any body region ≥ 4. The maximum GERtality score was 5 points. A mortality rate of 72.4% was calculated in patients with the maximum GERtality score. Mortality rates of 65.1 and 47.5% were encountered in patients with GERtality scores of 4 and 3 points, respectively. The area under the curve (AUC) of the novel GERtality score was 0.803 (GTOS: 0.784; RISC-II: 0.879). The novel GERtality score is a simple and feasible score that enables an adequate prediction of the probability of mortality in polytraumatized geriatric patients by using only five specific parameters.

## 1. Introduction

The elderly population increases worldwide and subsequently the number of geriatric trauma patients rises as well [1]. Geriatric patients require special medical attention due to the higher risks for mortality and morbidity related to frailty, reduced physiological compensation mechanisms after trauma, polypharmacy and preexisting comorbidities, both in high-energy trauma cases as well as in low-energy trauma situations [2,3,4,5,6].

Prediction-model based outcome scores are useful tools for judging patients’ status and to guide medical decision making. Especially in trauma, there is a need for adequate (mortality) prediction models to optimize post-resuscitation triage and the determination of initial therapy until transfer to the intensive care unit (ICU) in severely injured patients. Several trauma outcome scores have been developed in which patients’ age is also addressed. The RISC-II (Revised Injury Severity Classification II) and the newly published GTOS (Geriatric Trauma Outcome Score) seem to predict mortality in elderly poly-traumatized patients quite accurately. However, these scores highly rely on Injury Severity Score (ISS) judgments, which are known for their suboptimal inter-observer reliability [7,8,9]. Unlike the GTOS, the RISC-II was not specifically developed and validated for mortality prediction of the elderly severely injured patient, but is considered to be the most accurate prediction model for severely injured patients in German speaking countries. The Geriatric Trauma Outcome Score is composed of the following parameters: patient’s age, the ISS and red blood cell transfusion requirements, whereas more factors, 15 in total, are required to calculate the RISC-II score [10]. Thus, the GTOS system includes less factors, which has practical benefits; however, the RISC-II score has been shown to be more accurate [11]. The aim of the current study was to develop a feasible and accurate novel score (the GERtality score) which combines simplicity with high accuracy for the prediction of in-hospital mortality in geriatric trauma patients.

## 2. Experimental Section

### 2.1. The TraumaRegister DGU^®^

The TraumaRegister DGU^®^ of the German Trauma Society (Deutsche Gesellschaft für Unfallchirurgie, DGU) was founded in 1993. The aim of this multi-center database is the pseudonymized and standardized documentation of severely injured patients.

Data are collected prospectively in four consecutive time phases from the site of the accident until discharge from hospital: (A) Pre-hospital phase, (B) Emergency room and initial surgery, (C) Intensive care unit and (D) Discharge. The documentation includes detailed information on demographics, injury pattern, comorbidities, pre- and in-hospital management, course on intensive care unit, relevant laboratory findings including data on transfusion and the outcome of each individual. The inclusion criterion is admission to hospital via emergency room with subsequent ICU/ICM (intensive care medicine) care or reaching the hospital with vital signs and dying before admission to ICU.

The infrastructure for documentation, data management, and data analysis is provided by AUC—Academy for Trauma Surgery (AUC—Akademie der Unfallchirurgie GmbH), a company affiliated to the German Trauma Society. The scientific leadership is provided by the Committee on Emergency Medicine, Intensive Care and Trauma Management (Sektion NIS) of the German Trauma Society. The participating hospitals submit their data pseudonymized into a central database via a web-based application. Scientific data analysis is approved according to a peer review procedure laid down in the publication guideline of TraumaRegister DGU^®^.

The participating hospitals are primarily located in Germany (90%), but a rising number of hospitals in other countries contribute data as well (at the moment from Austria, Belgium, China, Finland, Luxembourg, Slovenia, Switzerland, The Netherlands, and the United Arab Emirates). Currently, approx. 33,000 cases from more than 650 hospitals are entered into the database per year.

Participation in TraumaRegister DGU^®^ is voluntary. For hospitals associated with TraumaNetzwerk DGU^®^, however, the entry of at least a basic data set is obligatory for reasons of quality assurance [12].

In order to gain data for the development of the new GERtality score, data from the TraumaRegister DGU^®^ from 1 January 2008 to 31 December 2017 were used.

The present study is in line with the publication guidelines of the TraumaRegister DGU^®^ and registered as TR-DGU project ID 2017-048.

### 2.2. Inclusion and Exclusion Criteria

The aim of this study was to develop a new mortality prediction model for severely injured geriatric patients. We excluded all patients below the age of 65 years, non-European hospitals, and patients with minor trauma (maximum Abbreviated Injury Score (AIS) of 1 or 2 without admission to the ICU). Patients with missing data regarding blood transfusion, as well as transfer in or early transfer out of the hospital, were also excluded from this study. Therefore, we selected all patients aged ≥ 65 years with an AIS of 2 or less who required intensive care treatment and all patients with an AIS of 3 or more from the TR-DGU. The following parameters were included: patients’ age, sex, ISS, maximum Abbreviated Injury Score (AIS) [13], pre-hospital and in-hospital diagnostics, initial and further treatment, trauma characteristics and the patients’ outcome.

### 2.3. Statistical Analysis

In the first step, patients’ data were dichotomized and the specific odds of all relevant variables (age, sex, American Society of Anesthesiologists (ASA) [14], trauma mechanism, Glasgow Coma Scale (GCS), maximum AIS, PRBCs (Packed Red Blood Cells) given prior to ICU admission, systolic blood pressure ≤ 90 mmHg) for in-hospital mortality were calculated in a univariable way. This was performed due to an expected mortality of at least 25% or more for every single parameter. Secondly, relevant variables were added to the new GERtality score and subsequently compared to the RISC-II, GTOS, maximum AIS, ISS and age by conducting a receiver operating characteristic (ROC) analysis.

## 3. Results

**Figure 1:**
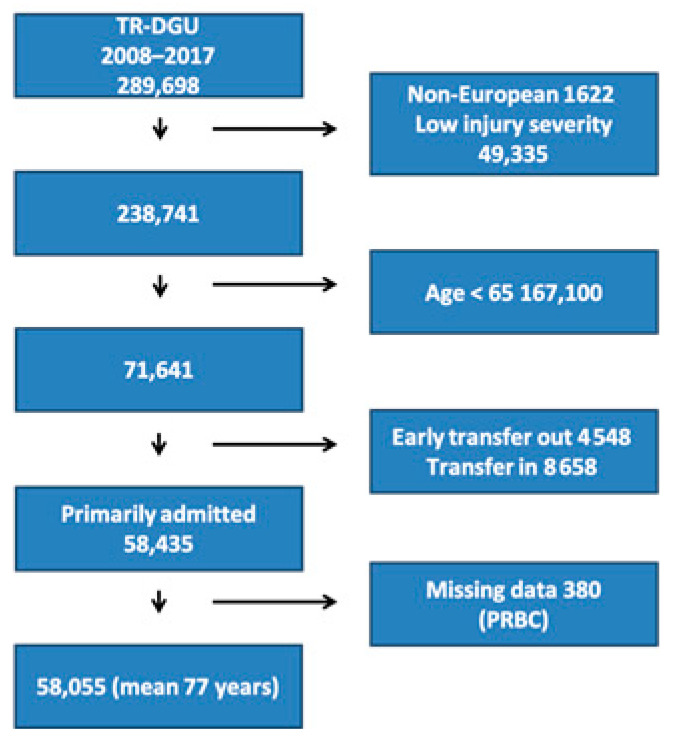
Inclusion flow of selected patients from the TraumaRegister DGU^®^ (TR-DGU). PRBC, Packed Red Blood Cells.

The TR-DGU included 289,698 patients from 2008 to 2017, of which 58,055 patients met the inclusion criteria. A PRISMA flowchart is provided in Figure 1. The mean age of all patients was 77 years, and 58% were males. Baseline characteristics are shown in Table 1.

GERtality score development:

We analyzed different parameters as sole predictors for in-hospital mortality. In a first step, relevant aspects with known prognostic relevance were defined: age, concomitant diseases, severity of head injury, relevant other injuries, and bleeding. Within each subarea, potential predictors were considered and compared. For continuous measures, cut-off values were derived to reach a mortality of ~30% or more. The final decision for a certain criterion was based on a multivariate odds ratio (OR) > 2.0 (Table 2).

The following patient specific parameters were suitable for the new GERtality score:

- Age ≥ 80 years
- Maximum AIS in any body region ≥4
- PRBCs received prior to admission to the ICU
- ASA ≥ 3
- GCS < 14

**Figure 2:**
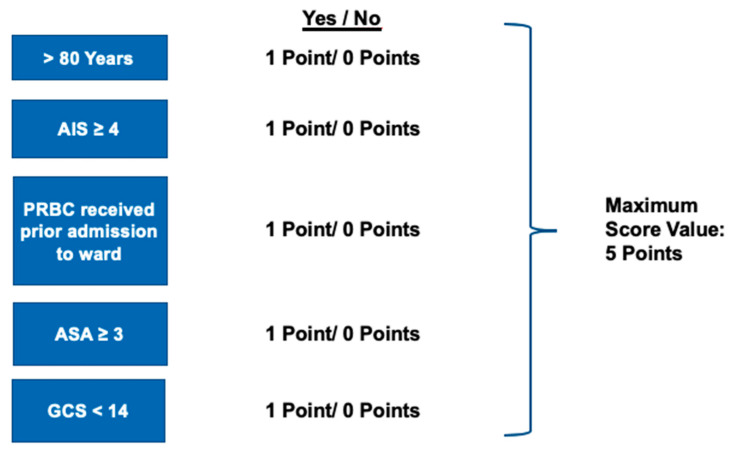
Relevant variables for the GERtality score. ASA, American Society of Anesthesiologists, GCS, Glasgow Coma Scale.

To calculate the new score, each finding, if present, adds up one additional point to the GERtality score. Thus, the new score ranges from 0 to 5 points (Figure 2).

**Figure 3:**
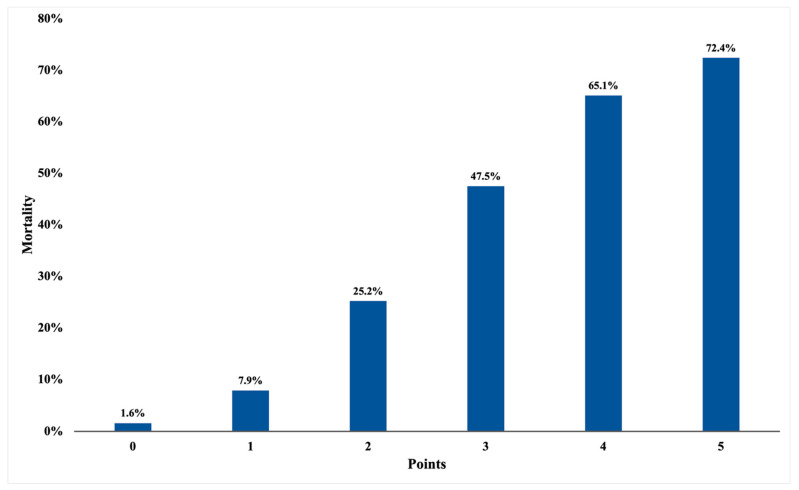
Observed in-hospital mortality rate based on GERtality score calculation.

The maximum GERtality score showed an in-hospital mortality rate of 72.4% compared to 65.1% with patients scoring 4 points on the GERtality score and 47.5% with a total score of 3 points. The mortality with a score of 0 was 1.6% (Figure 3).

**Figure 4:**
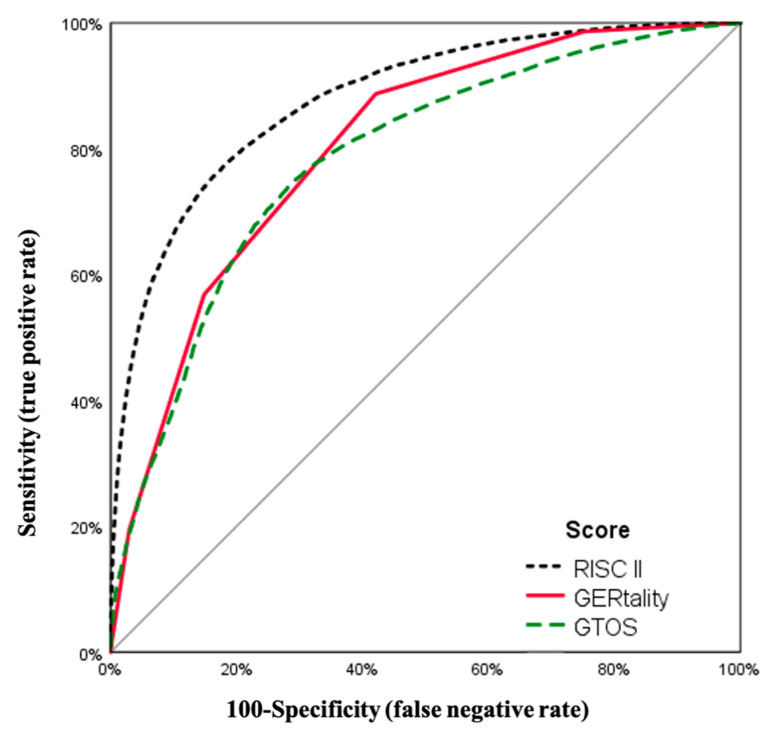
ROC (receiver operating characteristic) analysis.

GERtality score comparison:

The final ROC analysis of our patient collective showed an AUC (area under the curve) for the new GERtality score of 0.803 (CI (confidence interval) 0.799–0.807). The complex RISC-II score with its 15 variables showed an AUC of 0.879 (CI 0.876–0.883), whereas the Geriatric Trauma Outcome Score had an AUC of 0.784 (CI 0.780–0.789). Individual variables showed an AUC of 0.772 (CI 0.767–0.776) for the maximum AIS score, 0.753 (CI 0.748–0.757) for the Injury Severity Score, and 0.633 (CI 0.627–0.638) for age (Figure 4).

## 4. Discussion

Trauma scoring systems are important instruments for the optimization of clinical decision making, the determination of outcome and the standardization of clinical studies [15,16]. Several successful trauma outcome scores have been developed in the last few decades, such as the Trauma Injury Severity Score (TRISS), APACHE-II-Score, Revised Trauma Score (RTS) and the Revised Injury Severity Classification (RISC) Score [17,18,19,20]. However, extrapolation to the geriatric population has limitations and, therefore, to date, only a few feasible scores for the prediction of mortality in geriatric trauma patients exist. To our knowledge, only two scoring systems were explicitly developed for mortality prediction after trauma in the geriatric population: namely, the new Geriatric Trauma Outcome Score (GTOS) and the very recently published Elderly Mortality after Trauma Score (EMAT) [21]. Unfortunately, we were not able to evaluate the EMAT Score because the registry data do not include all parameters used in the scoring system. The well-established Revised Injury Severity Classifications-Score II (RISC-II), although not especially developed for geriatric patients, is believed to also calculate mortality in elderly patients the most adequately and is considered the gold standard in trauma outcome prediction. The GTOS uses the patient’s age, ISS and PRBCs for estimating the mortality of geriatric patients using the following rather intricate formula to calculate the possibility of death:

age + (2.5 × ISS) + 22 (if given PRBCs)

The GTOS predicts a chance of mortality of 50% with a score of 177 and a chance of 99% with a score of 310 [10].

The RISC-II, which was introduced in 2014, requires 15 different variables to predict mortality adequately [11]. The RISC-II, among other scoring systems, is believed to predict mortality the most accurately. Originally, this scoring system was not developed for the mortality prediction of geriatric patients, and with its carefully adjusted and weighted 15 variables, it is difficult to be calculated at the bedside. 

The EMAT score contains two scoring models: the quick elderly mortality after trauma score, which should be used at the initial presentation of the patient, and the full EMAT score for calculation after radiological evaluation. The qEMAT score can be calculated with eight variables, including systolic blood pressure, pulse rate higher than 120 bpm or lower than 50 bpm, GCS, penetrating injury, congestive heart failure, liver cirrhosis and chronic renal failure, whereas the fEMAT requires 26 variables to be calculated. In this mortality prediction model, each positive variable adds up points (e.g., systolic blood pressure < 90 mmHg = 17 points and heart rate below 50 bpm = 7 points) and can then be calculated by a mobile application which is provided freely by the authors. One of the limitations of this scoring system is that it was developed and validated using a geriatric population (>65 years of age). However, age was not used as an independent factor for mortality. The EMAT also does not address severe bleeding due to trauma as a leading cause of death in severely injured patients and, as a result, the need for blood transfusions [22]. Furthermore, we believe that the EMAT is not suitable for the European population since the incidence of penetrating traumatic injuries is much lower than in the U.S. [23]. In addition, in order to calculate the EMAT, full patients’ history, including co-morbidities, has to be provided, which often is not possible in the event of acute trauma [24].

Therefore, we aimed to develop a new scoring system which is easy and fast to calculate at site, but still predicts mortality in geriatric patients adequately.

In the collective of 58,055 patients used in this study, the AUC for the accuracy of mortality prediction of the GTOS was 0.784 and 0.879 of the RISC-II, whereas the novel GERtality score showed an accuracy of 0.803. These findings show that the RISC-II score is a highly accurate prediction score, but also has severe practical limitations which affect its feasibility. The RISC-II score combines a total of 15 different patient related variables which are rather unhandy to calculate on site. The GTOS, on the other hand, is relatively easy to calculate as it only uses three variables (age, ISS and received PRBS), but it had a slight disadvantage in accuracy towards the GERtality score. Furthermore, the RISC-II was made for post hoc calculation in databases and would require a computer, while the GERtality score intends to provide a simple point system to gain a quick and simple overview shortly after the admission of the patient.

It is well known that the parameters age, ISS and GCS are positive predictors for death [25,26]. In a study from a western European trauma ICU investigating the changes in outcome of severe trauma patients over a period of 15 years, age, hemorrhagic shock, GCS and the ISS were positive predictors of death [27]. Age, as a variable, combines an age-related decrease in immune defense as well as age-related comorbidities [28,29,30,31,32,33]. Frailty syndrome contributes to increased vulnerability in geriatric patients after severe trauma [34]. Generally, the treatment of unstable geriatric trauma patients does not differ a lot from non-geriatric trauma patients, but due to associated postoperative morbidities and complications in frail patients, for example, diagnostic laparoscopy should be preferred over open diagnostic laparotomy in hemodynamically stable patients [35]. Frailty is defined as clinically recognizable declines in the physiologic reserve of multiple organ systems as well as a decline in coping mechanisms for everyday life stressors [36,37]. This definition suggests that frailty is not defined by chronological age, but studies have shown a clear correlation between increasing FI (Frailty Index) and age in Europeans. However, frailty is a stronger predictor for mortality than chronological age [37].

In order to address comorbidities, we used the ASA classification as a variable in the GERtality score. As anticipated, this study shows that there is a strong association between age over 80 years and the probability of death after trauma. It may be interesting to focus on specific factors related to trauma mortality in octogenarians in further studies. 

It is also known that outpatient anticoagulants are associated with a higher likelihood of PRBC transfusions [38]. One of the leading causes of death in trauma patients is hemorrhage related irreversible shock [22]. This is underlined by the current study as an association between the need for blood transfusion and the probability of death in elderly severely injured patients was found. 

To sum up, the novel GERtality score combines the five most important variables associated with death after trauma in the elderly: age above 80 years, GCS < 14, maximum AIS ≥ 4, need for blood transfusion prior to ICU admission, and ASA ≥ 3. The newly developed score might help improve quality assurance, identifying the early need for transfusion/coagulation correction and decision making on further treatment for the polytraumatized geriatric patient. Before a decision on treatment, knowledge of calculated mortality odds can be advantageous, especially in usually complex ethical questions, which arise in geriatric patients frequently. 

There are some limitations present in this study. In general, the quality of registry data is considered inferior due to lacking data verification. Furthermore, we were unable to calculate the frailty index, which is considered to be a good predictor for mortality in geriatric patients. The TR-DGU does not document the patient’s Frailty Index, and as a consequence, we decided to utilize the chronological age in combination with the ASA Score in our dataset. 

As mentioned above, outpatient anticoagulant medication has a strong correlation with PRBC transfusions. Unfortunately, the use of anticoagulants was not assessed in this dataset because it was introduced as a novel parameter in the registry since 2015. In this study, we did not yet validate the developed score. 

## 5. Conclusions

The new GERtality score seems to be a feasible and adequate in-hospital mortality prediction model for severely injured geriatric trauma patients. The score includes only five easily assessable patient variables, which makes it practical and simple to calculate. Further studies should validate the novel GERtality score on different datasets. 

## Figures and Tables

**Table 1:** Basic data of 58,055 geriatric trauma patients.

Measurement	Unit	Value
Age	years	77.2/77 (7.6)
Male sex	*n* (%)	33,483 (57.8%)
Injury Severity Score (ISS)	points	19.2/17 (11.8)
Number of injuries	*n*	4.2/4 (2.6)
Penetrating trauma	*n* (%)	1426 (2.6%)
Mechanism: traffic	*n* (%)	19,910 (35.1%)
Mechanism: low fall (<3 m)	*n* (%)	25,218 (45.0%)
Mechanism: high fall	*n* (%)	7727 (13.6%)
Severe head injury (AIS ≥ 3)	*n* (%)	26,504 (45.7%)
Treated on intensive care unit	*n* (%)	51,166 (88.1%)
Ventilated on ICU	*n* (%)	22,486 (38.7%)
Length of stay in hospital	days	16.6/12 (16.7)
Hospital mortality	*n* (%)	12,969 (22.3%)

Continuous measurements are presented as mean/median (SD). AIS, Abbreviated Injury Scale, ICU, intensive care unit.

**Table 2:** Mortality rates and odds ratios of specific variables.

Variable	Subgroups	No. of Patients	Mortality Rate	Odds Ratio (OR)	95% CI of OR
Age	≥80 years <80 years	21,810 (38%) 36,245	31.5% 16.8%	2.27	2.18–2.36
Max. AIS	4 or more 2–3	25,924 (45%) 32,131	38.7% 9.2%	6.25	5.97–6.54
Blood transfusion	yes no	4813 (8%) 53,242	43.4% 20.4%	2.99	2.81–3.17
ASA	3/4 1/2	20,235 (41%) 28,269	29.1% 16.4%	2.09	2.00–2.18
GCS	3–13 14–15	22,559 (41%) 32,719	42.2% 8.8%	7.61	7.27–7.89

ASA, American Society of Anesthesiologists, GCS, Glasgow Coma Scale, CI, confidence interval.

## Data Availability

Data is accessible on reasonable request.

## References

[1] World Health Organization *Ageing and Health*; 2020. https://www.who.int/news-room/fact-sheets/detail/ageing-and-health.

[2] Hukkelhoven C.W.P.M., Steyerberg E.W., Rampen A.J.J., Farace E., Habbema J.D.F., Marshall L.F., Murray G.D., Maas A.I.R. (2003). Patient age and Outcome FOLLOWING Severe Traumatic brain Injury: An Analysis of 5600 patients. J. Neurosurg..

[3] Demetriades D., Sava J., Alo K., Newton E., Velmahos G.C., Murray J.A., Belzberg H., Asensio J.A., Berne T.V. (2001). Old age as a criterion for trauma team activation. J. Trauma.

[4] Joseph B., Zangbar B., Pandit V., Kulvatunyou N., Haider A., O’Keeffe T., Khalil M., Tang A., Vercruysse G., Gries L. (2014). Mortality after trauma laparotomy in geriatric patients. J. Surg. Res..

[5] Con J., Friese R.S., Long D.M., Zangbar B., O’Keeffe T., Joseph B., Rhee P., Tang A.L. (2014). Falls from ladders: Age matters more than height. J. Surg. Res..

[6] Peterer L., Ossendorf C., Jensen K.O., Osterhoff G., Mica L., Seifert B., Werner C.M.L., Simmen H.-P., Pape H.-C., Sprengel K. (2019). Implementation of new standard operating procedures for geriatric trauma patients with multiple injuries: A single level I trauma centre study. BMC Geriatr..

[7] Champion H.R., Copes W.S., Sacco W.J., Lawnick M.M., Keast S.L., Bain L.W., E Flanagan M., Frey C.F. (1990). The Major Trauma Outcome Study: Establishing national norms for trauma care. J. Trauma.

[8] Maduz R., Kugelmeier P., Meili S., Döring R., Meier C., Wahl P. (2017). Major influence of interobserver reliability on polytrauma identification with the Injury Severity Score (ISS): Time for a centralised coding in trauma registries?. Injury.

[9] Pothmann C.E.M., Baumann S., Jensen K.O., Mica L., Osterhoff G., Simmen H.-P., Sprengel K. (2018). Assessment of polytraumatized patients according to the Berlin Definition: Does the addition of physiological data really improve interobserver reliability?. PLoS ONE.

[10] Zhao F.Z., Wolf S.E., Nakonezny P.A., Minhajuddin A., Rhodes R.L., Paulk M.E., Phelan H.A. (2015). Estimating Geriatric Mortality after Injury Using Age, Injury Severity, and Performance of a Transfusion: The Geriatric Trauma Outcome Score. J. Palliat. Med..

[11] Lefering R., Huber-Wagner S., Nienaber U., Maegele M., Bouillon B. (2014). Update of the trauma risk adjustment model of the TraumaRegister DGU: The Revised Injury Severity Classification, version II. Crit Care.

[12] Deutsche Gesellschaft für Orthopädie und Unfallchirurgie (2019). Traumaregister DGU^®^.

[13] Haasper C., Junge M., Ernstberger A., Brehme H., Hannawald L., Langer C., Nehmzow J., Otte D., Sander U., Krettek C. (2010). The Abbreviated Injury Scale (AIS). Options and problems in application. Unfallchirurg.

[14] Doyle D.J., Garmon E.H. (2019). American Society of Anesthesiologists Classification (ASA Class).

[15] Abu-Hanna A., Lucas P.J. (2001). Prognostic models in medicine. AI and statistical approaches. Methods Inf. Med..

[16] Laun R.A., Schröder O., Schoppnies M., Röher H.D., Ekkernkamp A., Schulte K.M. (2003). Transforming growth factor-beta1 and major trauma: Time-dependent association with hepatic and renal insufficiency. Shock.

[17] Wagner D.P., Draper E.A. (1984). Acute physiology and chronic health evaluation (APACHE II) and Medicare reimbursement. Health Care Financ. Rev..

[18] Boyd C.R., Tolson M.A., Copes W.S. (1987). Evaluating trauma care: The TRISS method. Trauma Score and the Injury Severity Score. J. Trauma.

[19] Champion H.R., Sacco W.J., Copes W.S., Gann D.S., Gennarelli T.A., Flanagan M.E. (1989). A revision of the Trauma Score. J. Trauma.

[20] Lefering R. (2009). Development and validation of the revised injury severity classification score for severely injured patients. Eur. J. Trauma Emerg. Surg..

[21] Morris R.S., Milia D., Glover J., Napolitano L.M., Chen B., Lindemann E., Hemmila M.R., Stein D., Kummerfeld E., Chipman J. (2020). Predictors of elderly mortality after trauma: A novel outcome score. J. Trauma Acute Care Surg..

[22] O’Reilly D., Mahendran K., West A., Shirley P., Walsh M., Tai N. (2013). Opportunities for improvement in the management of patients who die from haemorrhage after trauma. Br. J. Surg..

[23] Lustenberger T., Talving P. (2016). Focus on challenges and advances in the treatment of patients with penetrating injuries. Eur. J. Trauma Emerg. Surg..

[24] Howard B.M., Kornblith L.Z., Conroy A.S., Burlew C.C., Wagenaar A.E., Chouliaras K., Hill J.R., Carrick M.M., Mallory G.R., Watkins J.R. (2015). The found down patient: A Western Trauma Association multicenter study. J. Trauma Acute Care Surg..

[25] Randolph A.G., Guyatt G.H., Richardson W.S. (1998). Prognosis in the intensive care unit: Finding accurate and useful estimates for counseling patients. Crit. Care Med..

[26] Matthes G., Seifert J., Bogatzki S., Steinhage K., Ekkernkamp A., Stengel D. (2005). Age and survival likelihood of polytrauma patients. “Local tailoring” of the DGU prognosis model. Unfallchirurg.

[27] Di Saverio S., Gambale G., Coccolini F., Catena F., Giorgini E., Ansaloni L., Amadori N., Coniglio C., Giugni A., Biscardi A. (2014). Changes in the outcomes of severe trauma patients from 15-year experience in a Western European trauma ICU of Emilia Romagna region (1996–2010). A population cross-sectional survey study. Langenbecks Arch. Surg..

[28] Broos P.L.O., D’Hoore A., Vanderschot P., Rommens P.M., Stappaerts K.H. (1993). Multiple trauma in elderly patients. Factors influencing outcome: Importance of aggressive care. Injury.

[29] Broos P.L., D’Hoore A., Vanderschot P., Rommens P.M., Stappaerts K.H. (1993). Multiple trauma in patients of 65 and over. Injury patterns. Factors influencing outcome. The importance of an aggressive care. Acta Chir. Belg..

[30] Dzankic S., Pastor D., Gonzalez C., Leung J.M. (2001). The prevalence and predictive value of abnormal preoperative laboratory tests in elderly surgical patients. Anesth Analg..

[31] Frankenfield D., Cooney R.N., Smith J.S., Rowe W.A. (2000). Age-related differences in the metabolic response to injury. J. Trauma.

[32] Knies R.C. (1996). Assessment in geriatric trauma: What you need to know. Int. J. Trauma Nurs..

[33] Leung J.M., Dzankic S. (2001). Relative importance of preoperative health status versus intraoperative factors in predicting postoperative adverse outcomes in geriatric surgical patients. J. Am. Geriatr. Soc..

[34] Chen X., Mao G., Leng S.X. (2014). Frailty syndrome: An overview. Clin. Interv. Aging.

[35] Di Saverio S., Birindelli A., Podda M., Segalini E., Piccinini A., Coniglio C., Frattini C., Tugnoli G. (2019). Trauma laparoscopy and the six w’s: Why, where, who, when, what, and how?. J. Trauma Acute Care Surg..

[36] Fried L.P., Hadley E.C., Walston J.D., Newman A.B., Guralnik J.M., Studenski S., Harris T.B., Ershler W.B., Ferrucci L. (2005). From bedside to bench: Research agenda for frailty. Sci. Aging Knowl. Environ..

[37] Romero-Ortuno R., Kenny R.A. (2012). The frailty index in Europeans: Association with age and mortality. Age Ageing.

[38] Ang D., Kurek S., McKenney M., Norwood S., Kimbrell B., Barquist E., Liu H., O’Dell A., Ziglar M., Hurst J. (2017). Outcomes of Geriatric Trauma Patients on Preinjury Anticoagulation: A Multicenter Study. Am. Surg..

